# Impact of Recurrence on Functional Independence in Stroke Patients Treated in a Convalescent Rehabilitation Hospital

**DOI:** 10.7759/cureus.72658

**Published:** 2024-10-29

**Authors:** Yoshihiro Kanata, Yuki Uchiyama, Saya Iwasa, Satoko Matsushima, Yuta Tauchi, Tetsuo Koyama, Kazuhisa Domen

**Affiliations:** 1 Department of General Medicine and Community Health, Hyogo Medical University Sasayama Medical Center, Tanbasasayama, JPN; 2 Department of Rehabilitation Medicine, Hyogo Medical University Sasayama Medical Center, Tanbasasayama, JPN; 3 Department of Rehabilitation Medicine, Hyogo Medical University, Nishinomiya, JPN; 4 Department of Rehabilitation Medicine, Nishinomiya Kyoritsu Neurosurgical Hospital, Nishinomiya, JPN

**Keywords:** fazekas scale, functional independent measure (fim), recurrent strokes, rehabilitation, stroke outcome

## Abstract

Purpose: Outcome prediction is a crucial component of rehabilitation in post-stroke patients. However, only a few studies have focused on the influence of stroke recurrence on outcomes in these patients. This study aimed to determine the impact of stroke recurrence on functional independence using data from a convalescent rehabilitation hospital.

Materials and Methods: The study included stroke patients who were admitted to our convalescent rehabilitation hospital. Data were collected on age, stroke recurrence, and number of days between stroke onset and transfer to our facility. Scores for the motor component of the functional independence measure (FIM-motor) and stroke impairment assessment set (SIAS-motor) were obtained at admission and discharge. Multivariate regression analysis was performed using the FIM-motor score at discharge as the dependent variable. Stroke recurrence was used as the independent variable, with age, days from onset to transfer, and FIM-motor and total SIAS-motor scores at admission entered as covariates. To explore the impact of the size of deep white matter lesions from prior strokes, we used the Fazekas scale to classify the recurrent cases into subgroups (0-1 or 2-3) and compared the FIM-motor scores at discharge between them.

Results: After adjusting for the above-mentioned covariates, stroke recurrence emerged as a statistically significant predictor of a reduced FIM-motor score at discharge. Furthermore, in patients with recurrent stroke, those with larger deep white matter lesions had a significantly lower FIM-motor score at discharge.

Conclusions: Stroke recurrence was found to be an independent predictor of a reduced FIM-motor score. Moreover, in patients with recurrent stroke, larger deep white matter lesions were associated with further reductions in the FIM-motor score. These findings underscore the negative impact of recurrent stroke on functional independence in stroke patients.

## Introduction

Stroke is a leading cause of disability among the elderly population worldwide [1]. Patients who have experienced a stroke often develop hemiplegia and/or cognitive impairments, which significantly impact their ability to perform activities of daily living (ADL) independently [2]. Rehabilitation is widely implemented from the early stages after stroke onset to facilitate restoration of functional independence [3], and accurate outcome prediction is crucial when planning an appropriate rehabilitation regimen [4]. For example, in the context of ADL, training programs for patients who are not expected to regain independent gait focus primarily on self-care activities such as eating, grooming, and dressing. In contrast, for patients who are anticipated to fully recover their locomotive functions, more challenging activities, such as stair-climbing, may become potential goals of the rehabilitation process.

A growing body of research has focused on the prediction of stroke outcomes. Some studies have used neuroimaging techniques [5,6], while others have used machine learning methodologies [7,8]. However, few studies have specifically examined the influence of stroke recurrence on outcomes in stroke patients [9,10]. According to a recent review, the rate of recurrence of ischemic stroke ranges from 5.7% to 51.3% [9], which highlights the importance of incorporating stroke recurrence in outcome prediction studies for greater real-world applicability. In stroke recurrence cases, patients were affected in functional independence more severely than in first-ever cases [10]. This study aimed to better characterize the impact of stroke recurrence on functional independence by standardizing the admission characteristics of patients with both initial and recurrent strokes. Using data from a convalescent rehabilitation hospital, the study investigated how stroke recurrence affects functional independence in the post-stroke period.

## Materials and methods

This study is based on a retrospective case-control survey of post-stroke patients who were admitted to our convalescent rehabilitation facility. The data analyzed were retrospectively collected for the period between November 2018 and March 2023. All patients received physical, occupational, and speech therapy sessions for up to 180 minutes per day, seven days per week during their hospital stay. The types of interventions, frequency, and duration of rehabilitation therapy provided were in accordance with the recommendations of the Japanese guidelines for the management of stroke [11].

Eligibility criteria included the ability to walk and independence in ADL in the local community before stroke onset (modified Rankin Scale ≤2) [12] and no evidence of dementia. Consistent with our previous studies on stroke outcome, individuals with subarachnoid hemorrhage or lesions in the cerebellum or brainstem were excluded from the final analysis [13,14], as were those who required acute medical intervention for conditions such as angina pectoris, gastrointestinal disorders, and fractures.

The protocol was approved by the Institutional Review Board of Hyogo Medical University (No. 4807). Informed consent for participation in this study was obtained using the opt-out method via the hospital’s website.

We collected data from the sampled individuals using the functional independence measure (FIM) [15]. The FIM is a tool that is widely used to evaluate independence in activities of daily living (ADL) and includes 13 motor items and five cognition items. Each item is scored on a 7-point scale (1, total assistance; 7, complete independence). Total scores for FIM-motor (range, 13-91) and FIM-cognition (range, 5-35) are commonly used in stroke rehabilitation. The severity of hemiparesis was evaluated using the motor component of the Stroke Impairment Assessment Set (SIAS-motor) [16]. This tool evaluates five components, namely, function at the arm, finger, hip, knee, and ankle, with each rated on a scale from 0 to 5. In this study, the overall severity of hemiparesis was quantified by calculating the total SIAS-motor score. The FIM and SIAS were assessed every 2 weeks throughout the hospitalization in our convalescent rehabilitation ward. Discharge was decided when the growth of the FIM-motor score reached its plateau. In this study, we used the scores at admission to and discharge from our convalescent rehabilitation hospital. We also collected demographic and clinical information, including age, sex, type of stroke (hemorrhagic or ischemic), history of previous stroke (recurrence), number of days between stroke onset and transfer to our rehabilitation facility, and total hospital stay (including acute care). The history of previous strokes was determined through medical interviews with patients. This methodology is commonly adopted in stroke studies conducted in Japan, as stroke diagnoses are typically made using magnetic resonance imaging (MRI) or computed tomography under Japan’s government-regulated health insurance system, which covers all residents.

We also performed a subgroup analysis of recurrent cases to determine the impact of previous strokes on functional independence outcomes. In this analysis, deep white matter lesions were assessed quantitatively on fluid-attenuated inversion recovery (FLAIR) images obtained by MRI. The Fazekas scale, which was developed to standardize the visual assessment of white matter hyperintensities observed on MRI [17], was applied to patients who underwent MRI during hospitalization. In this study, two experienced physiatrists independently scored patients using the Fazekas scale based on the FLAIR images. Any discrepancies were resolved by discussion until a consensus was reached. To simplify the analysis, we categorized deep white matter lesion scores on the Fazekas scale as 0-1 points or 2-3 points [18].

In the primary analysis, we compared demographic and clinical data, FIM-motor and SIAS-motor scores, and total hospital stay between patients with first-ever stroke and those with recurrent stroke using the t-test or chi-squared test as appropriate. For the main analytic procedures, we employed multivariate regression analyses using the forced entry method because these variables are known to affect functional independence outcomes [9]. The target values were the FIM-motor score at discharge and total hospital stay, which are considered to be important indicators of the efficacy of rehabilitation treatments in the Japanese healthcare system [19,20].

In these analyses, we assigned the following numerical values to the categorical variables: 0 for first-ever stroke and 1 for recurrent stroke. These values were used as explanatory variables. Age, days between stroke onset and transfer to our rehabilitation hospital, and FIM-motor and total SIAS scores at admission were included as covariates. For further analysis, we compared two subgroups classified according to the Fazekas scale. In this comparison, patient demographics and clinical data, including total hospital days and FIM-motor and SIAS-motor scores, were used as dependent variables. These analyses were performed using the t-test and chi-squared test as appropriate. All statistical analyses were performed using the JMP software package (SAS Institute Inc., Cary, NC). A p-value of <0.05 was considered statistically significant.

## Results

**Figure 1:**
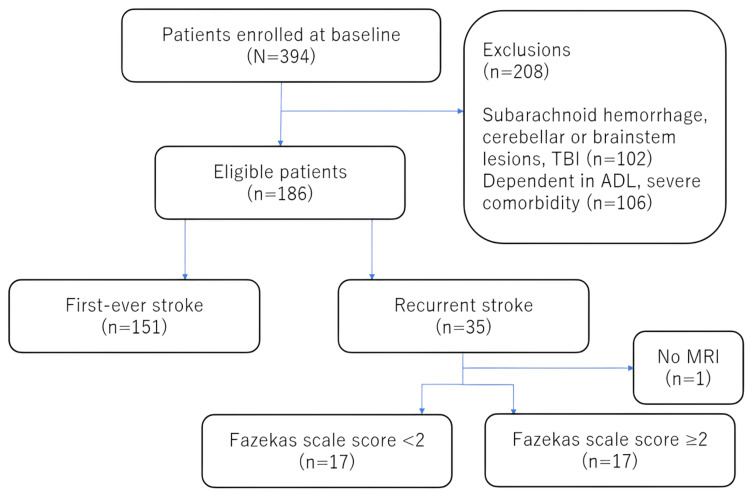
Diagrammatic sketch of the screening process ADL: activities of daily living; MRI: magnetic resonance imaging; TBI: traumatic brain injury.

The procedure used to screen patients for eligibility to participate in the study is shown in Figure 1. Of 394 stroke patients admitted during the study period, 186 met the eligibility criteria (first-time stroke, n=151; recurrent stroke, n=35). Among the patients with recurrent stroke, the Fazekas scale score was <2 in 17 patients, ≥2 in 17, and unknown in 1 case because of the lack of availability of MRI-FLAIR images.

The patient characteristics are shown in Table 1. Patients with recurrent stroke were slightly older, had a longer interval between stroke onset and admission to our convalescent rehabilitation hospital, and had lower FIM-motor and SIAS-motor scores at admission. However, these differences were not statistically significant. The potential influences of these non-significant differences were further examined through the multiple regression analyses presented below. In contrast, FIM-motor and SIAS-motor scores were significantly lower at discharge in patients with recurrent stroke. These patients also had a significantly longer hospital stay.

**Table 1:** Patient demographic and clinical characteristics The data are shown as the median [interquartile range] or as the number as appropriate. Statistically significant findings are shown in bold (p<0.05). F: female; FIM-motor: motor component of the functional independence measure; H: hemorrhagic; I: ischemic; M: male, SIAS-motor: motor component of the stroke impairment assessment set.

	First-ever stroke (n=151)	Recurrent stroke (n=35)	p-value
Age (years)	76 [67‐83]	77 [68‐87]	0.896
Sex (M/F)	86/65	25/10	0.110
Type of stroke (H/I)	59/92	10/25	0.240
Days from onset	26 [18‐37]	32 [21‐44]	0.169
FIM-motor at admission	39 [20‐58]	26 [15‐52]	0.109
SIAS-motor total at admission	15 [7‐21]	11 [5‐19]	0.056
Total hospital stay (days)	117 [74‐171]	159 [101‐181]	0.027
FIM-motor at discharge	79 [56‐87]	54 [23-83]	0.002
SIAS-motor total at discharge	19 [11‐23]	13 [6‐19]	0.012

Table 2 shows the results of the multivariate regression analysis for the FIM-motor score at discharge. Whether the stroke was first-ever or recurrent emerged as a significant predictor of the FIM-motor outcome. Consistent with the findings shown in Table 1, recurrent stroke was strongly associated with a lower FIM-motor score at discharge, as was older age, a longer interval between stroke onset and admission, and lower FIM-motor and SIAS-motor scores at admission.

**Table 2:** Results for FIM-motor at discharge obtained by multivariate regression analysis. Statistically significant findings are shown in bold (p<0.05). FIM-motor, motor component of the functional independence measure; SE, standard error; SIAS-motor, motor component of the stroke impairment assessment Set; VIF, variance inflation factor.

	Estimate	SE	t	p-value	VIF
First-ever/recurrence	-8.704	3.013	-2.89	0.004	1.023
Age	-0.469	0.103	-4.55	<0.001	1.254
Days from onset	-0.202	0.059	-3.40	<0.001	1.116
FIM-motor at admission	0.522	0.085	6.15	<0.001	2.701
SIAS-motor total at admission	0.761	0.226	3.36	<0.001	2.518
Intercept	75.786	8.968	8.45	<0.001	-

Table 3 presents the results of the multivariate regression analysis for total hospital stay. Three factors, namely, days from stroke onset, FIM-motor score at admission, and total SIAS score at admission were identified to be statistically significant determinants of total hospital stay. In contrast with the results shown in Table 2, the findings for age and whether the stroke was first-ever or a recurrence did not reach statistical significance.

**Table 3:** Results for total hospital stay obtained by multivariate regression analysis Statistically significant findings are shown in bold (p<0.05). FIM-motor: motor component of the functional independence measure; SE: standard error; SIAS-motor: motor component of the stroke impairment assessment set; VIF: variance inflation factor

	Estimate	SE	t	p-value	VIF
First-ever/recurrence	2.931	6.408	0.46	0.648	1.023
Age	-0.256	0.219	-1.17	0.244	1.254
Days from onset	0.860	0.126	6.82	<0.001	1.116
FIM-motor at admission	-0.803	0.181	-4.45	<0.001	2.701
SIAS-motor total at admission	-3.072	0.481	-6.38	<0.001	2.518
Intercept	193.528	19.074	10.15	<0.001	-

Table 4 compares the 34 cases of recurrent stroke according to whether the Fazekas scale score was <2 or ≥2. There was no significant between-group difference in age, FIM-motor or SIAS-motor score at admission, or total length of hospital stay. However, the FIM-motor score was significantly lower at discharge in the group with larger deep white matter lesions.

**Table 4:** Demographic and clinical data for the 34 patients with recurrent stroke according to the size of the deep white matter lesions as estimated by the Fazekas scale score The data are shown as the median [interquartile range] or as the number as appropriate. Statistically significant findings are shown in bold (p<0.05). F: female; FIM-motor: motor component of the functional independence measure; H: hemorrhagic; I: ischemic; M: male; SIAS-motor: motor component of the stroke impairment assessment set

	Fazekas scale score <2 (n=17)	Fazekas scale score ≥2 (n=17)	p-value
Age (years)	72 [66.5‐89]	82 [67‐87]	0.574
Sex (M/F)	11/6	13/4	0.450
Type of stroke (H/I)	3/14	7/10	0.128
Days from onset	34 [18‐46]	25 [21‐40.5]	0.952
FIM-motor at admission	37 [18.5‐63]	20 [13.5‐35.5]	0.052
SIAS-motor total at admission	10 [4‐19]	13 [2.5‐19]	0.500
Total hospital stay (days)	122 [90.5‐169.5]	168 [114‐199]	0.238
FIM-motor at discharge	65 [43‐86.5]	37 [17‐66.5]	0.038
SIAS-motor total at discharge	15 [7‐21]	13 [5‐19]	0.303

## Discussion

This study investigated the impact of stroke recurrence on two primary outcomes, namely, the FIM-motor score at discharge from a rehabilitation hospital and the total hospital stay [21-24]. Simple two-group comparisons indicated that patients with recurrent stroke had a lower FIM-motor score at discharge and a longer hospital stay (Table 1). To explore the impact of stroke recurrence further, we performed multivariate regression analyses with adjustments for the potentially confounding factors reported in the literature (Tables 2 and 3) [21-24]. These factors included age, FIM-motor score at admission, days from stroke onset, and neurological factors such as the severity of hemiparesis as indexed by the gross total SIAS-motor score. After adjusting for these potential confounders, stroke recurrence emerged as an independent predictor of a lower FIM-motor score but not of total hospital stay. Furthermore, the impact of the previous stroke on the FIM-motor score was more marked in patients with larger deep white matter lesions (Table 4).

A number of studies have been published on stroke outcome prediction [5-8]. However, few have directly addressed the impact of stroke recurrence, partly as a consequence of the diverse nature of populations with a history of stroke. For example, patients with lacunar infarction often have no evident neurological sequelae [1]. In contrast, those with severe infarction, such as intracerebral hemorrhage, may not fully recover their ability to perform ADL or function in the extremities [25]. Therefore, there is a wide range of impairments within the population with recurrent stroke. To address this issue, our study focused on community-dwelling patients who could walk and were independent in ADL [13]. These criteria suggest minimal neurological sequelae after previous stroke. Indeed, the statistical comparisons shown in Table 1 indicate that our patients with recurrent stroke were comparable with those who had first-time stroke in terms of FIM-motor and SIAS-motor scores. The study design controlled effectively for differences in background characteristics between the two groups, making the impact of stroke recurrence on the FIM-motor score more evident after adjustment for confounding factors.

Beyond simple two-group comparisons (Table 1), this study used multiple regression analysis to adjust for potential confounding factors (Tables 2 and 3). The choice of explanatory variables is important when adjusting for potential confounding factors by multivariate regression. For example, the type of stroke (hemorrhagic or ischemic) could potentially influence the results. However, our previous studies found no significant association between the type of stroke and FIM-motor score or total hospital stay [13,26]. The FIM assessment system includes both FIM-motor and FIM-cognition components. We included FIM-cognition data in our preliminary analyses. However, the correlation between FIM-motor and FIM-cognition scores was found to be 0.704. To minimize concerns about multicollinearity, we decided not to include FIM-cognition in the final analysis. Similarly, the SIAS-motor assessment includes five components (two for the upper extremity and three for the lower extremity), all of which are strongly correlated. To address concerns related to multicollinearity, we used the sum of the motor component scores for SIAS-motor and limited the number of covariates, which were considered to be potential confounding factors for the outcome measurements.

In our simple two-group comparisons, the hospital stay was longer in the group with recurrent stroke (Table 1). However, multivariate regression analysis, which allows for adjustment of covariates, did not identify recurrence as an independent predictor of a prolonged total hospital stay post-stroke (Table 3). To some extent, this finding may reflect the study period in that we analyzed medical charts for stroke patients admitted between November 2018 and March 2023, a timeframe that coincided with the COVID-19 pandemic [27]. Patients staying in convalescent rehabilitation wards are often assessed for their ability to manage daily living at home by allowing them to return home for a short trial period of 2-3 days just before discharge. However, during our study period, there was a need for strict restrictions for such assessment for infection control purposes. Although the study was not explicitly designed to address such concerns, this practice may have influenced our discharge decisions and the total hospital stay.

We used the Fazekas scale score to assess the impact of deep white matter lesions attributable to previous stroke on outcomes in patients with recurrent stroke and found that the extent of deep white matter lesions was associated with the FIM-motor score at discharge (Table 4). We did not determine the precise location of the deep white matter lesions, which is important in that bilateral lesions caused by recurrent stroke may hinder functional recovery. However, systematic assessment of the location of white matter lesions, including the presence of multiple lesions, would be challenging in a retrospectively identified cohort of patients from a convalescent rehabilitation hospital [28,29]. Further studies are needed to clarify this issue.

One of the strengths of this study is its emphasis on stroke recurrence, which is an under-studied topic. The main reason why outcome studies on recurrent strokes have been less conducted than those on first-ever strokes lies in the complexity of their history, including the number of episodes, specific lesion sites, and the severity of pre-existing neural damage. Although accounting for such factors may be clinically important, it could reduce statistical power due to increased complexity. In this study, we did not include these factors, which may have contributed to clearer findings on widely recognized functional evaluation tools (FIM and SIAS) that provide dependable outcome measures.

This study has several limitations. First, our sample of recurrent stroke patients was independent in ADL with minimal neurological sequelae. As a result, the study excludes a significant subset of the stroke population with substantial neurological deficits post-stroke, potentially leading to an incomplete understanding of stroke recurrence outcomes across different severity levels. Second, our database included patients with severe cognitive impairment, such as Alzheimer's disease [30]. Dementia is a prevalent comorbidity among the elderly, and the exclusion of patients with dementia constrained the sample size in our study. Third, we excluded patients with subarachnoid hemorrhage and those with lesions in the brainstem or cerebellum because their symptoms, which include altered consciousness and ataxia, differ significantly from those observed in individuals with lesions in the supratentorial intramedullary region. Fourth, the MRI examinations were performed at various hospitals, so the imaging conditions were not standardized. However, despite these limitations, our findings indicate that the recurrence of stroke was an independent determinant of a lower FIM-motor score at discharge as a long-term functional outcome after stroke. Fifth, for clarity, the study was not designed to explore the underlying mechanisms behind the poorer outcomes in recurrent stroke patients, and it did not include adjustments for various factors, such as rehabilitation intensity and socioeconomic factors. Further studies are needed to address these issues.

## Conclusions

In this study, we examined the impact of stroke recurrence on outcomes after standardizing the admission characteristics of patients with initial and recurrent stroke. As a result, we identified recurrent stroke as an independent predictor of a lower FIM motor score at discharge. Furthermore, in recurrent cases, more extensive deep white matter lesions were associated with a further decline in the FIM-motor score. While it is empirically known that stroke recurrence has a significant impact on functional outcomes, the findings of this study suggest that even if the sequelae of the initial stroke are minor, the impact on outcomes at the time of stroke recurrence is substantial, and the size of deep white matter lesions influences the outcomes.
